# Skin Disease or ‘Blood’ Disease?

**Published:** 1896-08

**Authors:** M. B. Hutchins

**Affiliations:** Atlanta, Ga., Clinical Lecturer on Skin Diseases and Syphilis, Atlanta Medical College


ATLANTA

Medical and Surgical Journal.

Vol. XIII. AUGUST, 1896. No. 6.

LUTHER B. GRANDY, M.D., MANAGING EDITOR.

M. B. HUTCHINS, M.D., BUSINESS MANAGER.

ALEX. W. STIRLING, M.D., C.M., M.B. (Edin.), D.P.H. (Lond.), MANAGING EDITOR PRO TEM.

COLLABORATORS:
A. W. CALHOUN, M.D., LL.D., VIRGIL O. HARDON, M.D., FLOYD W. McRAE, M.D., AND DUNBAR ROY, M.D.

### ORIGINAL COMMUNICATIONS.

### "SKIN DISEASE OR 'BLOOD' DISEASE?"

By M. B. HUTCHINS, M.D., Atlanta, Ga.,
Clinical Lecturer on Skin Diseases and Syphilis, Atlanta Medical College.

A hard-headed old citizen who was once on the losing side in a political race, said "Nine-tenths of the people are fools." This was a harsh judgment, almost equal to David's "All men are liars"; and perhaps also hasty. However that may be, the question at the head of this article grates upon one who has studied all which is involved in its answer. If the patient meant to ask whether he had syphilis, or some other trouble with his skin, it would be easier to answer, and the questioner would stand in less danger of being placed in the "nine-tenths" class. The whole truth is, that he does not know what he means. One has an idea that there is a lot of offensive poison floating in his blood, and that his skin lesion, or lesions, is the poison worked out on the surface. Pustular diseases therefore indicate pyemia, a cancer—cancers swimming in the blood; the eruption of poison ivy lives in his blood for seven years, coming out in summer, hibernating the rest of the time; it never being possible to catch the disease every year. The comparison might be carried further *ad absurdum*, but it is better to inquire for the origin of the question and its meaning.

Undoubtedly physicians have told, and do yet tell, their patients that their skin disease is a manifestation of blood disorder, is "in the blood." The physician who does not know any better is excusable; the physician who doesn't know much better, and uses the phrase to becloud the patient's mind, is probably not much to be blamed—if he is hard up. But the physician who knows better, is not hard up, but just simply knows enough to know it is not "in the blood," without being able to diagnose the condition, yet holds to the patient, is not a friend to the dermatologist, and perhaps would be the first to blame him for taking a case in general practice.

Another, and more widely reaching cause of the ignorance so ludicrously displayed regarding this and many medical subjects lies in the advertisements and literature of patent medicines. Intelligent people who study and reason out religious, ethical, legal, or political subjects, swallow whole, without mental mastication, these advertisements. Poisoning naturally follows from indigestion and mal-assimilation of the matter taken in.

A more reasonable class than the first mentioned is composed of those who mean to ask if the disease is constitutional or local. The patient who says there is nothing the matter with him but his skin eruption receives my profound respect. If there is any defect in his health we find it out and treat it. I once told a lady, whose mother had eczema, that the only blood disease of which I knew was one people were ashamed of.

It is a wearisome task, like looking for a needle in a haystack, to go through a volume on skin diseases searching for "blood" or constitutional diseases. When you have counted them all you may possibly have used the fingers of one hand. What is a blood disease? Is it a disease of the blood, or a disease in the blood? Certain elements may be in the blood, which the secretions have failed to eliminate. Do they appear upon the skin always in the form of a certain well-defined lesion or eruption? Diseases of the blood must be diseases in which the blood's normal constituents are affected or altered.

Now, to bring the matter down to a more logical basis, are skin diseases constitutional or local? Also ask the same question as to the diseases which have been grouped to form the other specialties, and those treated by the general practitioner. Take eczema, the most common of the diseases of the skin, and let us see what we can find to justify any belief that it is a manifestation of blood disorder, or that it is a constitutional disease. The inquiry is most easily answered by the works on dermatology and the note-books of those who treat skin diseases. Eczema does not occur as a symptom of any general disease, neither does it occur vicariously for such disease. There are conditions of the general economy which may act as predisposing factors, and which may retard the cure of the disease, but is this not equally true of any other disease? Certain people, being exposed to a certain group of causes (to the physician perhaps unknown), develop an eczema, other people with apparently the same general conditions develop an acne, others one of the erythemata, rosacea, acne rosacea, perhaps boils or carbuncles—the cocci of pus finding a fit soil in the skin of lowered vitality—etc. There are many general as well as local diseases which affect only those especially susceptible. Every man does not get tuberculosis, at first a local disease; nor kidney disease, nor cataract, nor atrophic rhinitis, nor enlarged prostate, nor cancer, and so on *ad infinitum.* Specific germs have been found to be responsible for a number of diseases, as tuberculosis, leprosy, the pus diseases, typhoid fever, malaria, etc. Insomuch has the "blood" theory been attenuated.

There is no known germ of eczema, of "nasal catarrh," of bronchitis, of gastro-enteritis. The tubercle bacillus causes lesions of the larynx, bronchi, lung, intestine, lymphatic glands, joints, bones, testicles, skin, etc. The typhoid bacillus occurs in the lesions of typhoid fever, the lepra bacillus in leprous lesions, the anthrax bacillus in malignant pustule, pus organisms in purulent conditions, and so on.

Is a subacute laryngitis or a proctitis a "blood" disease? Will "sarsaparilla" cure them? Is chronic endometritis a "blood" disease or constitutional?

The simple truth of the matter is that we have certain germs known as the cause of certain general, internal, external, or local diseases, and that we yet have many diseases in the domain of medicine the cause of which is not known. It is absurd to say a skin disease is an evidence of "blood" or constitutional dyscrasia, then to say that such and such a disease in some other specialty or in general practice is local, *sui generis* or idiopathic. We have many skin diseases which need nothing but local treatment; we have many in the other specialties requiring only local treatment.

On the other hand, we have many diseases—cutaneous, ocular, arthritic, uterine, orthopedic—which yield the more readily to local treatment if all deviations from good health are corrected. Further, if a diagnosis is necessary in other diseases, why is it not in skin diseases? If proper local treatment is necessary in diseases of the rectum or ear, why not in diseases of the skin? The encouragement of the common belief that skin diseases are "blood" diseases, and need only internal treatment, is a weak yielding of moral courage to the exigencies of practice, and a refuge for ignorance.

Dermatologists are not purely localists, but are more expert in the treatment locally of skin troubles. The small matter of diagnosis again comes to the front here, for upon diagnosis depends prognosis and treatment; and the fewer cases of any kind which the physician must treat "on general principles," the better for his fame.

A word as to the other parties responsible for this "blood" idea. The majority of patients who go to a skin specialist have taken "blood" medicines, and have used the "*-curas*" and "*-ines*" in much greater quantity than they would have for any physician; yet they finally get to the dermatologist, as the blood seems not to have been purged of the *materies morbi*.

Syphilis is a constitutional disease—the most perfect in the list of "ills that flesh is heir to." It is met by every general practitioner and every specialist. They use the same drugs, each case requiring its special physician only according to the location of the disease; yet the most certain evidences of the disease are the skin symptoms, and these are always sought. Syphilis, then, is the nearest approach to a blood disease. If the man has not syphilis it is doing him a great wrong to say that he has it, or something so close akin to it as to be "in the blood."

Excepting the many skin diseases labeled neurotic simply as a cloak for ignorance, we still have far more of them attributable primarily to a neurotic cause than are traceable to the "blood." But does this qualify the neurologist to treat them? By no means. He would not know which ones were neurotic unless they were such as typified the nerve or brain disease. At the same time we find at one time a skin disease in a neurotic patient, again the same disease in a dyspeptic, again the same in a teething baby, again in a varicose leg, a whiskey drinker, a genito-urinary patient, or, finally, and as often as in all the others combined, in a patient otherwise perfectly well. Given, one hundred patients with, and one hundred patients without, skin diseases, and the one hundred *without* will show just as great deviation from normal health as the one hundred *with* the skin diseases. This is proven by my own notes of cases, and can be verified any day by any physician.

There are many skin diseases which are less trouble to keep than to treat, but the large majority are either disfiguring, uncomfortable, distressing, or dangerous. The latter cases belong as naturally to the dermatologist as an iritis to the oculist, an engorged uterus to the gynecologist, or an appendicitis to the surgeon.

Other specialists have traveled the same route and have gained, or are gaining, recognition. Of dermatology it might be said it is recognized as yet only in certain localities; but just as sure as the skin is one of the most extensive, most important, and most accessible parts of the body, is it that dermatology has not long to wait for recognition as a clear-cut, necessary, and useful specialty.

Pioneers have fought their way, step by step, though confronted by many hardship, until they or their successors have finished the work and left easy progress for others. So is it in dermatology: pioneers have opened the way in many localities, and are opening it in others, with no intention of abandoning the struggle until the recognition they demand is obtained.

